# Voice Synthesis Improvement by Machine Learning of Natural Prosody

**DOI:** 10.3390/s24051624

**Published:** 2024-03-01

**Authors:** Joseph Kane, Michael N. Johnstone, Patryk Szewczyk

**Affiliations:** 1 Cyber Security Cooperative Research Centre, Edith Cowan University, 270 Joondalup Drive, Joondalup, WA 6027, Australia; m.johnstone@ecu.edu.au (M.N.J.); p.szewczyk@ecu.edu.au (P.S.); 2 Security Research Institute, Edith Cowan University, Joondalup, WA 6027, Australia

**Keywords:** LSTM, prosody, linguistics, text-to-speech enhancement, machine learning, phonemes, spoken, MOS

## Abstract

Since the advent of modern computing, researchers have striven to make the human–computer interface (HCI) as seamless as possible. Progress has been made on various fronts, e.g., the desktop metaphor (interface design) and natural language processing (input). One area receiving attention recently is voice activation and its corollary, computer-generated speech. Despite decades of research and development, most computer-generated voices remain easily identifiable as non-human. Prosody in speech has two primary components—intonation and rhythm—both often lacking in computer-generated voices. This research aims to enhance computer-generated text-to-speech algorithms by incorporating melodic and prosodic elements of human speech. This study explores a novel approach to add prosody by using machine learning, specifically an LSTM neural network, to add paralinguistic elements to a recorded or generated voice. The aim is to increase the realism of computer-generated text-to-speech algorithms, to enhance electronic reading applications, and improved artificial voices for those in need of artificial assistance to speak. A computer that is able to also convey meaning with a spoken audible announcement will also improve human-to-computer interactions. Applications for the use of such an algorithm may include improving high-definition audio codecs for telephony, renewing old recordings, and lowering barriers to the utilization of computing. This research deployed a prototype modular platform for digital speech improvement by analyzing and generalizing algorithms into a modular system through laboratory experiments to optimize combinations and performance in edge cases. The results were encouraging, with the LSTM-based encoder able to produce realistic speech. Further work will involve optimizing the algorithm and comparing its performance against other approaches.

## 1. Introduction

TTS engines are powerful assistive tools to read content from computer screens for those that require assistance with reading or have visual impairments. TTS engines are ubiquitous, with computer systems using TTS engines to produce a synthetic, human-like voice which allows for a lower barrier to entry for human interfaces to computers by making the interaction a conversational request. In contrast to some TTS engines that are still identifiable as non-human, this study will demonstrate how a standalone algorithm can be applied to generated or recorded audio in order to improve the prosodic elements such that the audio is no longer identifiable as robotic.

One of the most iconic uses of a synthetic voice was for the late Stephen Hawking. While Professor Hawking never changed the sound of his synthetic voice, it was not because of a lack in advancement of technology [1]. Synthetic voices with recognizably friendly tones have also facilitated the growth in voice-activated digital assistants McCaffrey et al. [2], yet despite advancements in technology there continues to be a distinction between the human recorded voice and computer synthetic voices.

Humans are natural story tellers [3] and there is an art to an engaging and interesting oration. Monotonous speech is disengaging and the brain actively ignores [4] such speech. In the human voice, the melody, rhythm, intonation, and cadence changes based on the meaning of what is being said. The changing patterns of rhythm and intonation in speech are described as prosody by Arvaniti [5]; however, the intended meaning is not the only driver in fluctuations in rhythm or tone of speech—physiology adds an element of randomness to the human voice. The addition of key prosodic elements to synthetic voices could modify the robotic nature of such voices and facilitate natural-sounding synthesis. Increasing realism allows enhanced electronic reading applications, and improved artificial voices for those in need of artificial speech assistance. Enhanced computer-generated speech improves interactions for the visually impaired and enhancing the voice interface to a computer increases the utility of the computing device. A computer that can also convey meaning with audible announcements will also improve human-to-computer interactions and there is scope for improving encoding mechanisms for digitized voices over internetworks or repairing damaged recordings. Adding prosody to existing TTS can be likened to the colorization of black and white films, and similarly may also be used to enhance old audio recordings. This article explores the improvement of digital spoken audio from low to high fidelity with natural sound, by identifying and adding prosodic elements using deep learning neural networks.

In this study, we present the following contributions to TTS enhancements and linguistics:

- We demonstrate the automated extraction of key prosodic elements for analysis, and train multivariate LSTMs on these interdependent variables.
- The LSTMs produce predictions of each feature variable, which are then input to the original audio sample. Utilization of the LSTM is critical in order to create predictions from multivariate time-series data.
- We demonstrate that the prosodic feature values can then be adjusted with the output from the trained LSTM. The updated values in the audio output creates a more natural prosody in the speech output. This is a novel approach as we are directly updating the prosodic information in an audio sample, which fills a research gap described in Section 3.

The paper is structured as follows. Section 2 and Section 3 review relevant theoretical foundations with a survey of the state of the art. The experimental approach is described in Section 4. Section 5 presents and discusses the results. Finally, Section 6 concludes the work.

## 2. Background

The WHO (Geneva, Switzerland) suggests that one billion people around the world have an unresolved vision impairment, from moderately impaired to classified blind [6], suggesting a large group of people that may require assistance with screen reading. In America, the NIDCD (Bethesda, MD, USA) reports that 9.4 million adults in the USA report having had a problem with their voice that lasted over a week [7]. Computer speech algorithms can do more than just improve the aesthetics of computer usage—a capable digital voice assistant can read from the screen or speak for its user. Unfortunately, computer algorithms make human voices sound robotic. Robotic sounding voices can also manifest from humans speaking into digital devices such as mobile phones due to codec issues, simply because of the way a codec attempts to either fit the signal into the bandwidth available or fill gaps due to lost data.

There are varied use cases for text-to-speech conversion such as providing a voice for those unable to vocalize, improving audio quality over low quality links such as radio, or enabling call centers with realistic audio-assistive chat-bots. People who have lost their voice due to a tracheotomy or other ailments are reliant on a computerized voice for communication to enhance their interactions with others. Application in radio could also assist emergency services by enhancing conversation clarity—a problem when people are under stress—reducing re-transmission due to muffled speech and potentially saving lives. Law enforcement may be aided by having a computer take statements, if a digital voice assistant has an accent that matches the demographic of those giving a statement then more detail may be forthcoming. Similarly, psychologists may benefit by using a digital confidante for their clientele.

The issue of realistic text-to-speech conversion is further complicated if the source and destination are separated by, for instance, the cloud. The protocols in computer networks work primarily on a principle of packetization, where large quantities of data are split into small chunks (packets) and sent sequentially. When an analog signal is encoded digitally, it is passed through an algorithm called a codec. Computer networks are often designed to be fault-tolerant rather than reliable. When packets are transmitted, they may arrive out of sequence or become lost due to the transmission medium—for example, a wireless transmission may suffer interference. McKay and Masuda [8] demonstrated a loss in perceived audio quality by positioning a radio transmitter next to a Wi-Fi network; however, it was not clear how the interference affected the audio quality, just that it was perceived. Broom [9] went further and rather than a radio source introducing interference, directly controlled the network parameters to induce interference and used PESQ as an objective measure to identify the grade at which data packet loss is associated with perceived quality. PESQ is used to predict subjective MOS scores via direct comparison of audio before and after it has been transmitted across a potentially lossy network. Bandwidth and latency issues also result in a loss in audio fidelity, especially in codecs with limited compression capability [10]. Audio artifacts can also be introduced where the codec itself is overloaded by background noise; Verma et al. [11] investigated the use of noise canceling and echo reduction systems to differentiate signal from noise before the codec digitizes the signal. A high signal-to-noise ratio input into the audio codec enhances the efficacy of the codec, resulting in higher-fidelity audio being transmitted, received, and perceived.

## 3. Related Work

The evolution of TTS has its origins in the work of Christian Gottlieb Kratzenstein in 1779 [12], who created a mechanical voice using a reed organ that could be played to produce vowel sounds. Digital computers and associated technology allowed for the first computerized TTS, with early inroads into adding prosody focused more on the word boundaries and pitch [13]—based on defining the structure of phrases. The DECTalk synthesizer, made famous by Professor Hawking, was reviewed in 1984 by the New York Times as “usually understandable” [14] with its phonetic ruleset—which is a mapping of letters to phonemes, and dictionary lookups of words into component phonemes, to be processed by the phoneme module. The phoneme-to-voice module then creates the series of voiced and unvoiced sounds, for example, “do I sound human?” is found in the dictionary as “du aɪ saʊnd hju mən?” and each phoneme in turn is then played through the synthesizer, with the preprocessor detecting the question mark and adding pitch rise in the final word [15]. Apple Siri^®^ (Cupertino, CA, USA) continues on the theme of increasing granularity on the phoneme by utilizing a half-phones database that is searched by a DNN to recombine the half-phones into a series of phonemes that comply to the trained prosody rules for the required utterance [16]. More advanced mechanisms are being used to overcome the lack of naturalness in synthetic speech: Lei et al. [17] utilized sentiment analysis in order to improve the naturalness in Chinese speech generation; and rather than using defined phonemes, Microsoft [18] (Redmond, WA, USA) build on language model work to utilize tokens that an ANN has identified.

Juvela et al. [19] described a method for speech synthesis using a GAN derived from a mel-spectrogram. A GAN was used for waveform synthesis as it uses a feed-forward architecture capable of parallel inference, thus increasing the speed of operation—particularly important for real-time speech synthesis. They reported that their method achieved higher MOS values than the reference system, but acknowledged a quality gap between natural and generated acoustic synthesis. The MOS, or mean opinion score, is an industrial standard metric used to measure artifacts and intelligibility of codecs and voice transmission, as recommended by the International Telecommunications Union [20]. Juvela et al. [21] note that GANs are especially appealing for waveform synthesis for realistic speech generation, and created a GAN-excited linear prediction neural vocoder, called GELP, which synthesizes speech waveforms from mel-spectrogram features. GELP achieved higher MOS scores than a reference WaveNet system. The parallel inference used in the GELP architecture makes GELP much faster than WaveNet. The use of a GAN was extended by Jin et al. [22] with the development of a deep learning approach called FFTNet, based on fast Fourier transforms. They showed that it was possible to synthesize a natural-sounding audio waveform directly from a deep convolutional neural network and presented an improvement over its precursor, WaveNet [23], in terms of execution speed, thus facilitating real-time audio synthesis; and it was tested via an MOS test. Compression was explored as another way of extending GAN by Li et al. [24] who found benefit in compressing the dynamic range of the spectrum (a common technique in practical audio applications). They used a new spectrum GAN, termed S-GAN, and found that compression improved the generator’s training, at least with the open-source dataset “VoiceBank + DEMAND”.

A related problem was solved by Kons et al. [25], where a DNN-based system was trained using a single speaker and then used to produce a variety of external speaker voices. Similar to Hodari et al. [26], Kons et al. [25] used a variational autoencoder-based prosody generator. Their approach to prosody generation is interesting, where the prosody network contains three bi-directional LSTM layers and the fourth fully connected linear layer generates a four-dimensional prosody vector per TTS unit, consisting of the unit log-duration, start log-pitch, end log-pitch, and its log-energy. They tested the DNN subjectively using an MOS test. LSTM models [27] have proven strong capabilities and flexibility, with an advantage of LSTM ANN being the ability to contextualize dependencies in the ANN by adding a working memory to the algorithm. A working memory allows for an ANN to essentially bookmark crucial elements, which the ANN learns are relevant through adjustment of specialized gates during training. The LSTM working memory is suited, therefore, for prediction of sequencing and predicting time-series data. Jorge et al. [28] demonstrated the use of a bi-directional LSTM as a mechanism for speech recognition using trained vocabularies for recognition. Khandelwal et al. [29] described a bi-directional LSTM as being two LSTMs, each with input reversed from the other, and the output combined, in order to improve predictions. Leveraging spectrographic images and concatenated fractal dimensions, Atila and Şengür [30] utilized an LSTM to classify emotive sentences. Self-attention transformers are an alternative to LSTM, supporting parallelism and longer sequences (especially useful for translation requiring multiple word sequences) while requiring a shorter training time [31]. Liu et al. [32] extended the attention-module-enhanced LSTM with a temporal pool based on an SVM.

Natural-sounding synthetic voice elements are sought after in various ways. Barbulescu et al. [33] described a joint audio–video approach towards speaker-identity conversion. Of interest is their method for adding prosody by examining the local slope of source and target speech and using this parameter to estimate a speaker-specific rhythm of speech to create new speech. Karpagavalli and Chandra [34] conducted a review of automated speech recognition architectures. Whilst this review solves the opposite problem to this paper, the approaches identified are instructive. They categorize automated speech recognition into acoustic–phonetic, pattern-recognition, and artificial intelligence approaches. The first approach assumes there are distinct phonetic units in a spoken language. The pattern recognition approach appears to be a standard machine learning approach, while the final approach uses a “hybrid of the acoustic phonetic approach and pattern recognition approach” (their phrase). In early work by Mary and Yegnanarayana [35], it was suggested that the speech signal should be segmented into syllables, which are typically language-specific. They segmented speech into syllables using vowel onset points (VOPs)—the instant at which the onset of a vowel takes place in a syllable—and represented the fundamental frequency ($f_{0}$) contour by several parameters, including change in $f_{0}$, distance of $f_{0}$ from the VOP, amplitude, and duration. Similarly, Tan et al. [36] surveyed the landscape of TTS synthesis, and described the evolution of text-to-speech algorithms from early synthesizers to RNN-based DNNs, and identified a generalized flow from character/phoneme to output waveform in all cases. Ultimately, they seem to suggest using machine learning on phonetic units, which appears to be a reasonable way forward.

Hodari et al. [26] observed that many existing approaches operate on sentences—a sentence may be too large a ’chunk’ as complete sentences likely contain a variable number of prosodic phrases. They captured prosodic elements using several variations of autoencoders and evaluated the results qualitatively, where test subjects were presented with two renditions of the same sentence and asked to determine different intonation (or not). They noted that the subjects perceived some duration and loudness changes, though neither of these features were modified (which perhaps highlights the difficulty inherent in using subjective ordinal measures). Phoneme, half-phone, or quantized tokens are enhancements in the phonetic representation of speech, identifying increasingly accurate models of phonemes, but the synthesis of the human voice requires more than an accurate recording being replayed. Recent work by Wang et al. [18] encoded a speaker identification matrix, which contains speaker-identifiable encoded audio information, to then train a hybrid DNN-HMM, which comprises a DNN and a HMM. The trained model was then able to utilize a specified speaker’s voice and then convert input text into audio that closely resembles the input speaker. Whilst this appears a promising approach, Wang et al. [18] acknowledged that “We observe that some words may be unclear, missed, or duplicated in speech synthesis”. Syllable-level features were normalized to train a real-time emotion recognition classifier [37], enabled by targeted feature extraction from the training corpora.

Part of the reason that phonetic representation has gained depth but not breadth is the difficulty involved in describing the subtleties of spoken language. Arvaniti [5] described the nuances of some of the differences. For example, between paralinguistics and prosody—the emotion or intent is not itself prosodic and can be identified through other mechanisms such as facial gestures; however, prosodic features can be used to index this information—stressed vowels contain variations in amplitude or timing, and it is through social learning that when to use either is conditioned as a single understanding of a stressed vowel. Highlighting concerns of misunderstanding due to ambiguous usage of terms such as prosody, meter, and rhyme, Zhang and Song [38] described differences using poetry, and how prosodic performance also differs: With different performance types come differing rulesets. Prosodic elements cross cultural and linguistic divides, but while the prosodic elements are similar, their use may not be. For example, Ekpenyong et al. [39] defined Ibibio as a tonal language, whereby the pitch of a word changes its meaning–this is also the case for Mandarin; a common example is the word “ma”, which, depending on tone, can mean mother, horse, scold, or hemp. This is in contrast to English, where pitch can turn a statement into a question, or shift emphasis between the subject and object of a spoken phrase. English and Chinese prose and prosody were also evaluated by Zhang and Song [38], highlighting the difficulties in translation from the Chinese linguistic terms to accurately reflect prosody; drawing out the ambiguity of the common usage of prosodic descriptors, as also found in Arvaniti [5]. Human biology also affects speech. For example, elision [40] is a shortening of spoken words caused by the difficulty in moving mouth parts—literal tongue twisters, as the tongue cannot simultaneously be at the front and the top of the mouth—while another example is that pauses are inserted due to breath. Elision in Mandarin was explored by Wu et al. [41] by utilizing a decision tree and noted improvements in speech quality using MOS after contracting syllables. A speaker’s connected speech has unsystematic breath/speech duration and timing [42] and evidence suggests longer internal sentence planning leads to larger pauses for breath [43,44].

A research gap exists in the addition of prosody directly into synthesized English speech. Instead of being identified as a discrete element to be added to synthetic voices, the focus in other research seeks an accurate portrayal of a syllable almost from a recording and, therefore, using a human voice as a synthesized instrument—instead of synthesizing the output from the biological processes to emulate prosodic elements of human speech that come from the biology of humans. This is perhaps the reason that artificial voices continue to be identified as such—existing text-to-speech algorithms are essentially engines that play a human voice as an instrument of recorded phonemes, or create their own voiced phonemes from the amalgam of the training opus. Other research is limited to a single aspect of prosody: this research seeks to inject multiple prosodic elements into existing audio. Targeting existing audio, therefore, also lends itself to upscaling low-bandwidth or heavily compressed audio.

Machine learning is one solution to the addition of relevant (authentic) prosodic information to audio. It has been used successfully in other domains, such as network traffic analysis [45,46] and biometrics [47]. There are several pitfalls, however, as described by Johnstone and Peacock [48]. To overcome some of these issues, a variant of a recurrent neural network, called a long short-term memory or LSTM is attractive. LSTMs can process time-series data, such as audio. Unlike conventional neural networks, LSTMs aim to capture short-term relationships, which can be advantageous when dealing with subtle frequency changes, as can be found in natural speech.

## 4. Materials and Methods

The research approach is to extract identified key elements of prosody from the voice samples and train an LSTM utilizing these key elements. The same key elements are then extracted from a non-prosodic sample and the LSTM learning is used to transform the key elements from monotonous and unchanging into dynamic prosodic elements. The transformed elements are then reapplied to the non-prosodic sample. Praat, a well-known voice audio analysis tool that also has the capability to manipulate layers of audio samples, is used for both the key-element extraction and the application of the transformation to the non-prosodic audio sample. The audio sample is subdivided into syllables, with analysis being conducted on these syllables, with the chosen key elements for LSTM training being:

- $f_{0}$—the fundamental frequency of each voiced syllable;
- the amplitude/intensity of each voiced syllable;
- the duration of each voiced syllable;
- the position of the voiced syllable in the spoken phrase;
- the duration of the syllable as a percentage of the spoken phrase;
- the length of the pause before and after each syllable.

### 4.1. Research Design

The research design is a quasi-experimental design. The hypotheses are declared and proven by experimentation in a controlled environment. The research question to be explored is: Is it possible to add prosody to speech synthesis to simulate authentic human speech?

The main hypothesis is that prosodic features can be extracted and be used in a deep learning neural network in order for computer-generated spoken words to be authentically human sounding.

**Hypothesis 0.**  *An enhancing algorithm built with a neural network does not improve the prosody of monotonous spoken audio.*

**Hypothesis 1.**  *An enhancing algorithm built with a neural network can improve the prosody of monotonous spoken audio.*

### 4.2. Laboratory and Experiments Design

**Figure 1:**
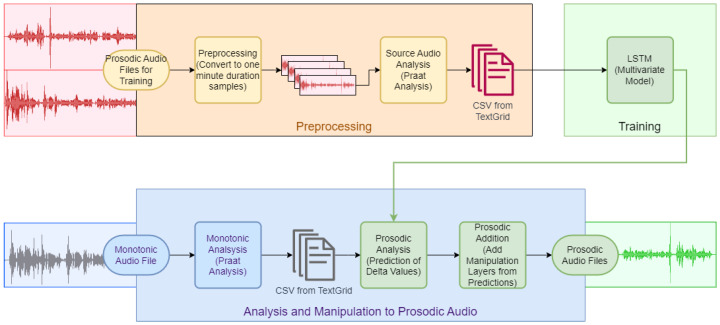
Prosody addition pipeline.

Processing of the data took place on a laptop that contained a single GPU, 16 CPU cores, and 32 GB RAM, with NVMe storage. Critically, NVMe storage reduces the latency which may occur from SAN-based storage to on-board, which improves performance in batch file processing. Local access to files and audio playback overcomes many of the challenges of using a cloud-based remote access approach. Initial experiments using cloud-based training proved non-competitive with respect to expenditure for equivalent experimentation time. TensorFlow and Keras were used as well as the following Python libraries: librosa, torchilbrosa, torch, praat-parselmouth, myprosody, mytextgrid, and pydub. Praat is used to identify the syllable by vowel detection, and pauses that have sound in them that are not vowel sounds are treated as consonants. Pauses that have no sound are treated as genuine pauses; for the purpose of this experiment the filled pauses are discarded and the genuine pauses before and after a syllable are recorded in the output for the LSTM training. The amplitude peak of each vowel sound is also recorded as the vowel maximum, which is used as a point location to add an anchor for the manipulation layers. The mid-point of each pause is calculated for a location to anchor duration manipulation. TED talks were used as the samples for long duration audio files for the training of the LSTM network. Eight hours of TED talks were downloaded and converted in the processing pipeline (Figure 1).

The three primary stages in the pipeline are “preprocessing”, “training”, and “analysis and manipulation to prosodic”. The first stage splits the long audio samples into a set of smaller audio files, each with a maximum one minute duration—shorter duration files were permitted into the training set. The second task in the first stage, entitled “source audio analysis”, consists of a Praat script which runs against each of these smaller samples to create a TextGrid (a tabulated text-based representation of key values extracted) that represents the voiced and unvoiced syllables. The TextGrid syllables are then further analyzed for the key elements, which are then extracted and exported to a temporary file to form the input to the LSTM.

An LSTM is selected, as using a multivariate LSTM for multidimensional interdependent time-series data in audio linguistics represents a novel approach. Traditional methods often struggle to capture the intricate dependencies within such complex datasets. However, by employing a multivariate LSTM, which can handle multiple input variables and learn time-based dependencies, we can better model the interplay between various linguistic features over time. This enables more accurate analysis and predictions.

In order to train the LSTM, in the second stage, “training”, the output from the “preprocessing” stage is input into multiple multivariate LSTMs, which are utilized to enhance accuracy and prevent the interdependent variables from affecting the training. Stochastic gradient descent was selected as the optimization function, and mean squared error as the loss function. The third and final stage, “analysis and manipulation to prosodic”, performs the same “source audio analysis” as before but instead of analyzing the source data files now analyzes the monotonic sample. The monotonic sample is a male voice reading from a novel with monotone and singular staccato rhythm. The monotonic analysis of the robotic file is then input into the trained LSTMs in the task “prosodic analysis”, which uses the trained LSTM to create delta values from the original key elements of the robotic sample analysis table to create predictions of these values. The final task, “prosodic addition”, uses the predicted values from the LSTMs to create new layers in Praat for intensity, duration (used by the values of the delta of the length of the syllable, and for the deltas of the pauses before and after), and a pitch manipulation layer, which adjusts the frequency. Merging the manipulation layers with the source audio creates a prosodic audio sample from the robotic audio file.

An LSTM can be modelled as univariate—a single-series input with a single-series output—or as a multivariate system with multiple series as inputs and one or more output series. As part of the framework, Keras initializes the LSTM with random values. In early experiments, it was discovered that due to the initial randomization of the ANN that a multi-output LSTM would not maintain zero values as zero, inadvertently affecting the accuracy. In experimentation, it was found that this initial randomized status of the neural network affects zero values, where these are not impacted for univariate experimentation. In order to build more accurate models, multiple many-input-to-single-output models are used in order to predict each of the targeted datasets. Due to the co-dependent input and output values this also allows for independent fine-tuning of each LSTM utilized.

**Figure 2:**
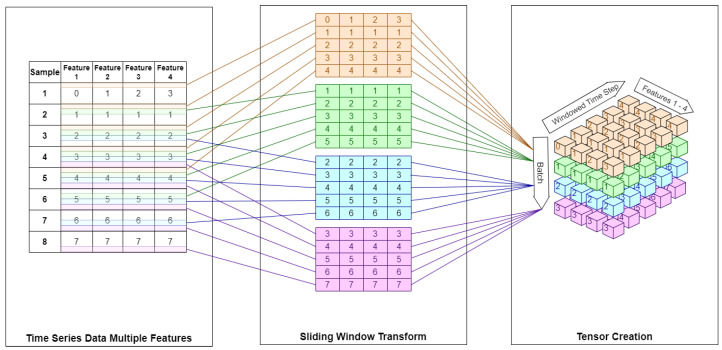
Sliding window multi-feature time series to tensor.

Initial experiments utilized time-series training based upon a fixed offset window of the first 60 elements as the source data, with the elements from 61 onwards being the target data. This was sufficient for univariate experimentation, but a general case was required to ensure scalability and additional testing of parameters, including changing the sliding windows of time-series data beyond a fixed number of 60 elements. The code pipeline was updated to add a function to convert an array into a sliding window multidimensional series from that array. The sliding window function takes parameters of the array passed to it and the number of steps (num_steps) to include in each sliding window. The input array is then iterated through, one element at a time, along its first axis, and the element and num_steps elements are then copied to an output array with an extra dimension, so that the output array contains a series of sliding windows of the input array. Utilizing this method allows for multidimensional inputs to be grouped together as interdependent inputs, and allows for a sliding window to be assigned to a single output variable. A visual representation of the sliding window is shown in Figure 2, which demonstrates the transformation from time-series data of multiple features to a sliding window, which is then converted into a 3D tensor.

The first part of Figure 2, designated “time series data, multiple features”, shows the multiple features as the columns, and each observation/sample as a row. For the example, the first observation shows the first feature’s value as 0, the second feature’s value as 1, the third feature’s value as 2, and the fourth feature’s value as 4; for simplicity, all features’ values are 1 in the second observation.

The middle section of Figure 2 shows a sliding window algorithm in operation; it creates a series of matrices, each with 5 elements—a sliding window size of 5. Each matrix contains entries that overlap with the matrices before and after it. For each matrix created, the startingRow is incremented by one, and the number of rows copied in from the time-series data is the sliding window size. The first matrix, therefore, represents n=5 and startingRow=1 copied from the time-series data, shown as an orange table with orange lines from the time-series data showing the origin of each row for clarity. The second matrix, shown by the green table with green lines to the time-series data, represents n=5 and startingRow=2 (having been incremented by one) samples copied from the time-series data. Multivariate LSTM models are more complex and require multidimensionality—in Keras the input must have a specific shape: A multidimensional object of the form [number of observations] × [length of input(time window size)] × [number of variables], and therefore, require a three-dimensional tensor to be passed to train the LSTM. The tensor creation is demonstrated in the third section of Figure 2 by layering each of the matrices created from the sliding window algorithm, creating the three-dimensional tensor.

An LSTM was trained for each of the required variables as a predicted output based on the input of all variables. The output predictions are:

- Delta of the duration of spoken syllable, in seconds;
- Delta of the pause before syllable, in seconds;
- Delta of the pause after syllable, in seconds;
- Delta of fundamental frequency ($f_{0}$) of spoken syllable, in Hz;
- Delta of intensity (amplitude) of spoken syllable, in dB.

**Figure 3:**
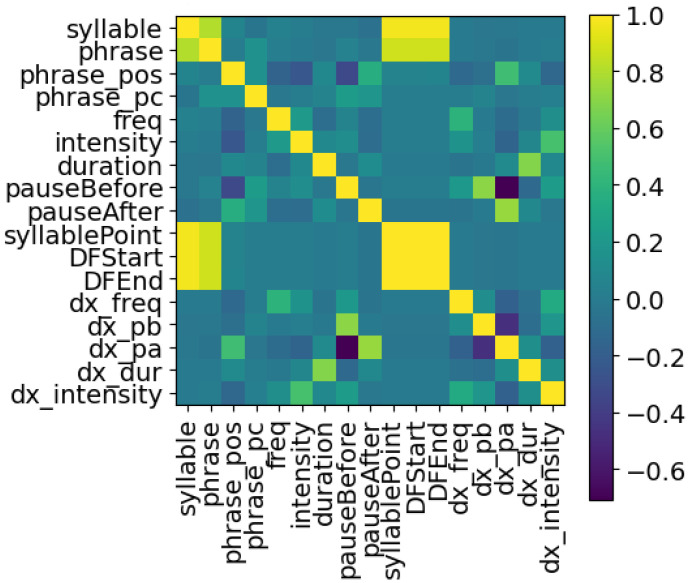
Pearson correlation coefficients between features.

The logic table of Table 1 explains which inputs were used to predict each target. Note that each target has its own trained LSTM. The phrase number, the size of the syllable within the phrase, and the syllable’s position within the phrase are used for all outputs. The target variables are themselves an input to other targets—this helps to improve the model, as if this was a multivariate output these variables would not be usable as input to the training. A Pearson correlation coefficient heatmap is shown in Figure 3. The “syllable point” variable is used for the recreation of the voice after training and is removed for the LSTM training: removing highly correlated data points is important to ensure the data points are orthogonal.

There is an LSTM-per-feature output prediction, resulting in five multivariate LSTMs; each is a unidirectional sequential LSTM. The number of layers and nodes per layer were investigated by recording the loss function by epoch: anything that was too ‘square’—in that the loss function recorded no further loss after just a few epochs—was disregarded due to overfitting, and gradients that did not appear to regress to a minimum were also discarded. The final model size is reported in Section 5.

The LSTMs provide a linear regression predictive model. In order to check the accuracy of the model, a multiple regression correlation using feature ablation was performed and is reported in Section 5.

**Figure 4:**
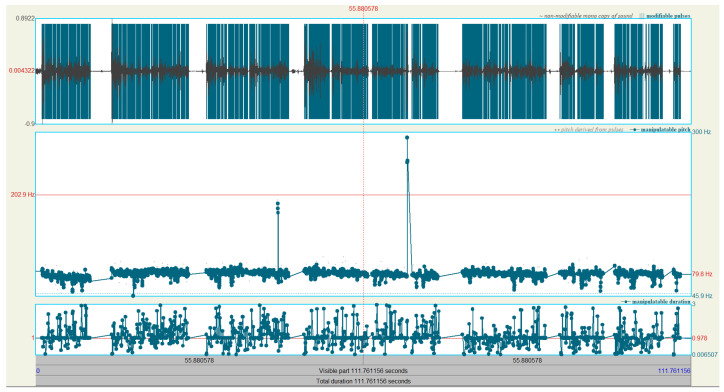
Praat manipulation.

**Figure 5:**
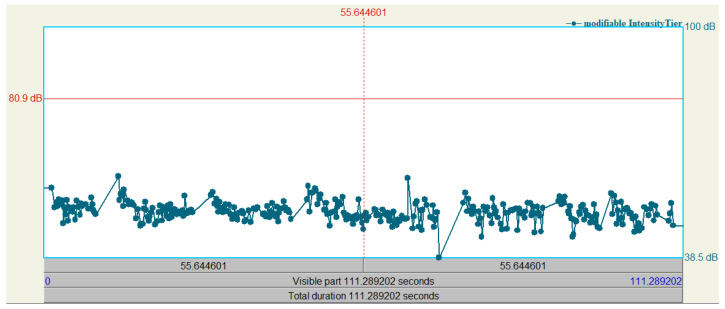
Praat intensity manipulation.

Praat manipulation layers are utilized to effect the LSTM predictions, these manipulation layers are titled “pitch tier”, “duration tier”, and “intensity tier” in the software. Figure 4 and Figure 5 show these tiers, with an example of values in each layer from the LSTM, as only the pitch tier is auto-populated. Figure 4 contains three window panes; the top pane displays a two-minute snapshot of the non-prosodic audio sample pre-manipulation, the *y*-axis represents amplitude, and the *x*-axis shows time.

In the middle pane—the pitch tier—the series of connected points represent frequency in the range of 50 Hz to 300 Hz—each point can be adjusted vertically to change the frequency, be removed, and additional points can also be created. Praat primarily uses autocorrelation to estimate the frequency points—the selected frequency range of 50–300 Hz highlights the use of band filtering in the Praat analysis.

The bottom pane is the duration tier and similarly has a series of connected points, the adjustment of which changes the speed of the audio sample at that point, and the integral of two connected points is used to calculate a new duration with linear interpolation for all points in between. A point at the start and a point at the end having a connecting line at a value of 2 has the effect of doubling the duration of the entire audio; a point at the start with a value of 0.5 and a value of 2 at the end has the effect of starting the audio by halving the time domain and increasing smoothly until it is stretched at the end. In order, therefore, to utilize LSTM predictions of the new time duration, a calculation of the ratio new_duration/old_duration is required.

Figure 5 shows a single pane with the intensity tier only, here the connected points set the intensity to a specific value. If using Praat in an interactive mode, the frequency layer is automatically populated from analysis of the audio file, and the duration and intensity tiers have no points; the automation creates each of these layers from the LSTMs’ predictions and utilizes the feature set of Praat to apply the changes to the original audio. The duration tier has three times as many points as the others as it has duration adjustments for each syllable, the pause before, and the pause after each syllable.

An automation script creates empty pitch, duration, and intensity tiers, then adds time points on each from the LSTM predictions. The pitch tier has a time point created per peak of each syllable, the value of each added point is the original frequency analysis with the delta as predicted applied.

The duration tier has a time point created for the center of each pause—before and after—and the amplitude peak of each syllable. Each of the time points on the duration tier has a value of the time calculation ratio such that the time for a pause or syllable is compressed or lengthened. The manipulation takes into account that shortening the duration increases the frequency, and so the pitch tier target is factored into the Praat manipulation. The intensity tier has a time point created per amplitude peak of each syllable, the value of each point is the predicted amplitude in decibels. Finally, the manipulation layers are then applied to the original; the intensity tier is added firstly, then the combined duration and frequency tier is applied.

## 5. Results

Initial experiments discovered a skew on the output voice predictions. The data values for frequency being used to train the LSTM included audio sources from both female and male voices, resulting in training an LSTM to create a target output that sounded like a deep-voiced woman, or a high-pitched man. A change in approach was enacted—to use the difference between frequency values (delta) as the target for the training; this change ultimately presented the desired behavior of the LSTM, as timing and frequency changes determine an authentic-sounding, melodic, prosodic voice. This approach was used for each value in each of the LSTM models created. In addition, Praat parameters were set to a specific upper and lower range—acting effectively as a band-pass filter—to ensure that the frequencies being captured for analysis were within the range of human spoken voice frequencies. The data preparation phase, therefore, had additional stages to ensure that the deltas are created as well as data cleansing.

Following the process depicted in Figure 1, the results are described in the following sub-sections: Analysis of source audio data, the LSTM training and audio processing, and analysis of the monotonous audio file. The experimental audio sample of a male voice speaking in monotone with singular rhythm was converted into prosodic audio using the LSTM prediction and Praat manipulation layers. The data analysis phase extracted:

- From the audio sample:
  – the syllable number;
  – the phrase number.
- Calculated the number of syllables in the phrase, and therefore:
  – the position in the phrase of the syllable (phrase_pos variable);
  – the percentage of the phrase (phrase_pc variable).
- Analyzed the voiced syllable frequency;
- Analyzed the voiced syllable intensity/amplitude;
- Calculated the pause before, the duration, and the pause after the voiced syllable.

### 5.1. Analysis of Source Audio Data

As described in Section 4, the source audio data were eight hours of TED talks from various male and female speakers.

Each talk was split into one-minute samples, each sample was then split into phrases. Each phrase is a grouping of syllables that are word or sentence fragments. As noted by Biron et al. [49], there is no broad consensus about the boundaries between phrases (which they call intonational phrases or intonation units), although they acknowledge that the boundaries are typically associated with slowing of the speech rate at the end of a phrase and a concomitant acceleration at the start of the following phrase. Braunschweiler and Chen [50] found that the mean of pauses in their training corpus was 210.7 ms and 393.1 ms for breathing pauses; an audio story read had pauses of 218.3 ms and breathing pauses of 352.7 ms. By examination, in our samples we determined that phrases were separated by time gaps under 300 ms and that gaps larger than 300 ms separated the groupings of syllables into the next phrase, as also found by Rahman and Tomy [51].

**Figure 6:**
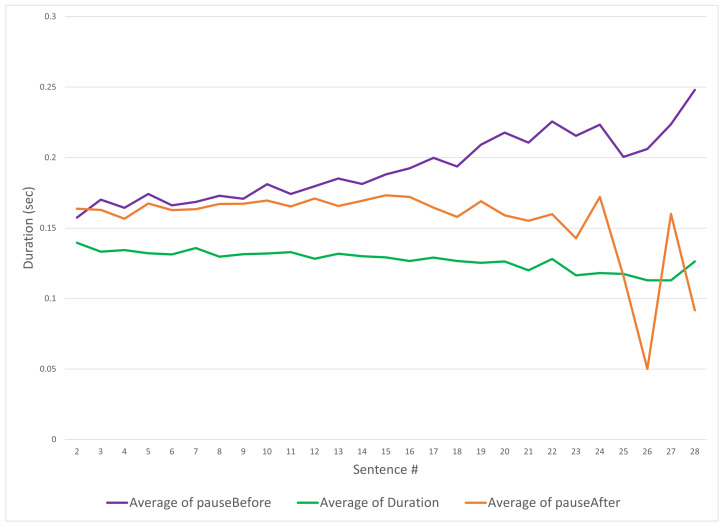
Duration of syllable vocalizations and pauses by phrase.

Section 4 mentioned that an important part of the data extraction from the source audio files was determining the position of the spoken syllable within the phrase (group of syllables). Figure 6 shows three duration elements of a spoken syllable in seconds: The syllable itself in green, and the pauses before (in purple) and after the syllable (in orange). The *y*-axis shows the duration in seconds and the *x*-axis represents the sequence number of the sentence. When reviewing the duration of each identified syllable from the source data in Figure 6, the average duration of each syllable decreases as the phrase number increases—this is understandable, as the samples are time limited to a maximum of one minute each, and with more phrases per sample it is expected that the duration must reduce.

The pauses before and after being correlating with the number of utterances highlights that this is a key element in the prosody being captured. Note that the pauses on either side of the spoken syllable appear to diverge (the orange and blue lines in Figure 6). The divergence between the “pause before” and the “pause after” is explained as being because the pause after is likely to be zero at the end of a sample, and similarly the pause before is zero at the beginning of a sample, as different samples end at different sentence numbers, the trend for the average value tends to zero. In Figure 6, the *x*-axis begins at the second sentence because the first syllable (being the initial syllable) has no “pause before”. It is important to keep these values to ensure that the LSTM differentiates the start and end of the samples. It is interesting to note the pause before increases with phrases spoken; as human speakers breathe the pauses’ increases can be explained by breathing. The interquartile range of the fundamental frequency of the audio samples has a median of 175 Hz for $f_{0}$.

**Figure 7:**
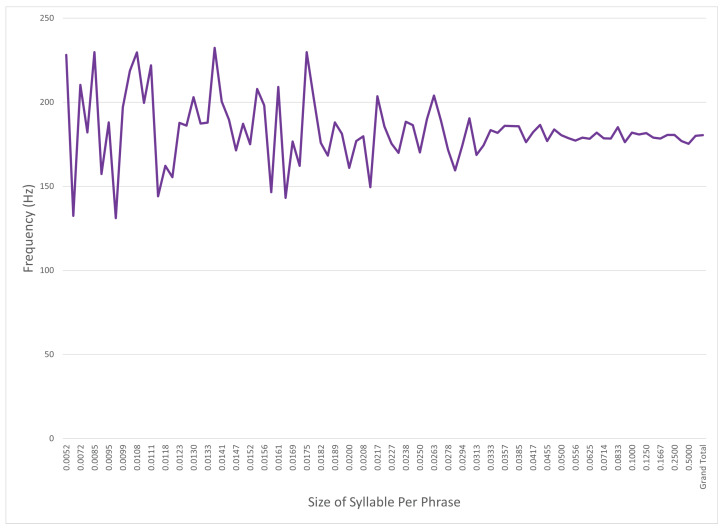
Average syllable frequency by percentage of phrase.

Figure 7 shows the average $f_{0}$ on the *y*-axis, while the *x*-axis has the relative size of the syllable compared to the phrase: For example, a size of 0.5 is a two syllable phrase, and a size of 0.125 is an eight syllable phrase. Figure 7 shows that syllables in the source data that are a larger portion of the phrase average occur at a higher frequency, while syllables that are smaller components of the spoken phrase have higher variance. This may be because of vocabulary differences between different speakers or perhaps intonation of shorter words naturally tends to high frequencies.

### 5.2. Training and Audio Processing

**Figure 8:**
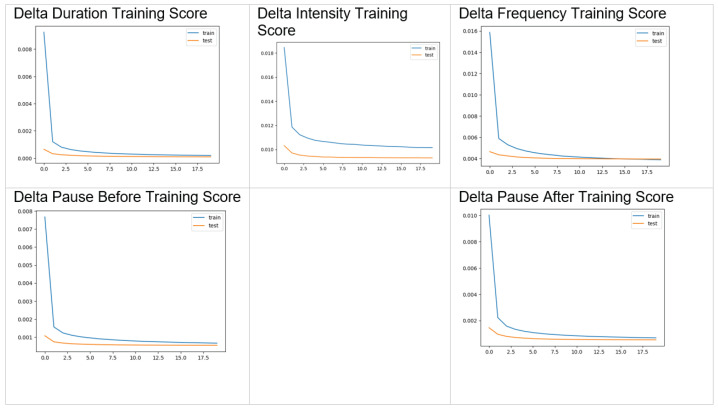
LSTM training scores.

The optimal training was found to be a single layer with 11 nodes, and a time window of 100 observations, allowing for time-efficient training. This created the smoothest curves for the valuation score loss, as depicted by the graphs in Figure 8. This is to ensure the model is neither overfitting nor spread across too many observations, such that it is predictive across all features being trained.

**Figure 9:**
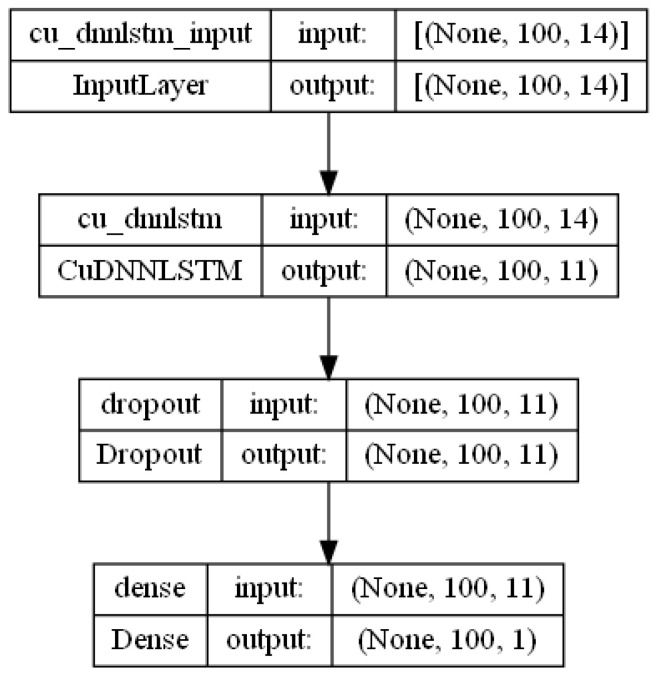
LSTM shape.

The blue lines in the graphs of Figure 8 represent the LSTM training data, including the dropout that is customary for the long short-term memory to forget yet to retain long term features. The orange lines represent the mean squared loss error in the regression for the test data, which notably does not include any dropout as it is a validation of the model. The flat lines in the images of Figure 8 indicate that the model is not regressing further. The optimal shape for the DNN was found to be relatively small, as depicted in Figure 9: An input shape of 100 samples × 14 features, a CuDNNLSTM layer with 11 units, a dropout layer set to 0.25, and a dense layer with 1 unit. The loss function was set to mean squared error, and the optimizer was set to sigmoid.

The lower the loss function score, the more accurate the model—it is clear that the model was less able to fit to the frequency/intensity, showing that on this smaller subset of sample data it appears to be closer to random than the other values. The training has outputs that resemble the source data but are not identical—this is the ideal, as otherwise the model would be overfitting. However, closer alignment would be preferable, and there may be additional fine-tuning to be undertaken. Following the third process depicted in the pipeline shown in Figure 1, an audio recording of a male reading a novel using a flat tone and singular-rhythm staccato syllable pronunciation was then processed by Praat to extract the key prosodic elements. The prosodic elements were then input to the multiple-trained LSTM to form predictions, as per the logic table in Table 1, and these predictions were imported into Praat a second time with the delta value to adjust the key elements to realize the prosodic transform. The delta values were added to the original values within a manipulation layer of Praat. The Praat layers are manipulations of the delta added to its related layer—dx_intensity maps to the intensity tier, dx_frequency maps to the pitch tier, etc. Finally, the non-prosodic audio sample was combined with the manipulation layers via Praat.

### 5.3. Analysis of Monotonous Non-Prosodic Audio Pre- and Post-Manipulation

Subjectively, it is evident that there is an improvement in prosody. The timing of the spoken audio is no longer staccato and the tone is no longer monotonous, but is subtly cyclical in its rhythm and the frequency of the spoken voice is no longer flat. This is an improvement from early manual experiments and much improved from the artificially female skew of the initial experiments. Objectively it is possible to perform the same audio analysis as was provided to the machine learning model to gain further insights. When comparing the pre- and post-manipulation of the non-prosodic spoken audio little has changed—despite the noticeable variation aurally.

**Figure 10:**
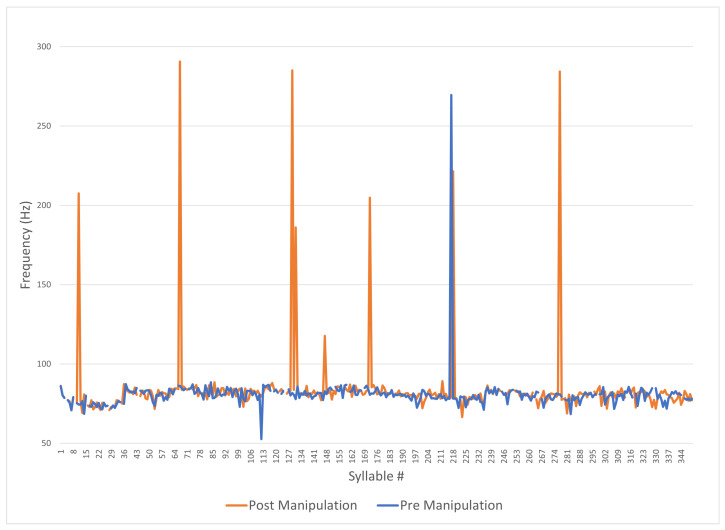
Praat pre- and post-manipulation (frequency).

Figure 10 shows the Praat analysis of a monotonous voice recording before and after manipulation, with $f_{0}$ ranging from 50 Hz to 300 Hz along the *y*-axis, and the *x*-axis showing each syllable number in its original sequence. The blue line represents the pre-manipulation audio and the orange line represents the post-manipulation audio. The shape of the original audio is loosely evident from the post-manipulation of the audio file and this is to be expected as it shows that the change to develop the model by using the delta of the values has allowed for additional prosody without losing underlying information. The post-manipulation line (in orange) clearly has seven high peaks compared to the single high peak of the original audio; however, at this resolution it is difficult to observe anything beyond these large spikes. Clearly, something has been added to the monotonous sample, but the subtleties between 75 and 85 Hz are hidden.

**Figure 11:**
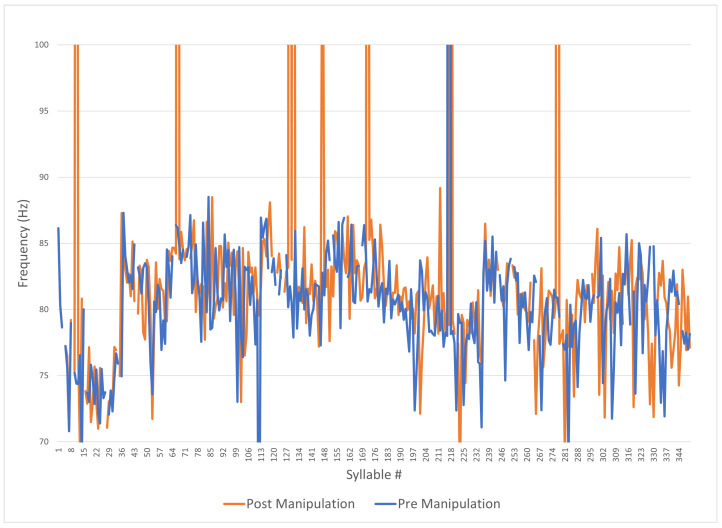
Praat pre- and post-manipulation (frequency zoomed).

Figure 11 shows the same data as Figure 10 with the *y*-axis expanded between 70 Hz and 100 Hz to more clearly demonstrate certain artifacts of the wave form. The frequency appears centered at 80 Hz in both the pre- and post-prosodic addition, with some artifacts at higher frequencies (which may be harmonics of the fundamental). Figure 11 shows that the transformed audio has a slightly wider frequency range but more high-frequency artifacts.

Band-filtering the initial Praat analysis has remediated many of the artifacts in the audio post-manipulation that are too fast, or too high pitched. The addition of fine-tuning with a notch filter using a Gaussian filter on the mel-spectrum will be explored in additional research to ensure that pops and fast-forwarded elements are tuned out, such that the perception is that of an authentic human speaking and not that of an artificially externally adjusted system working on a recording.

**Figure 12:**
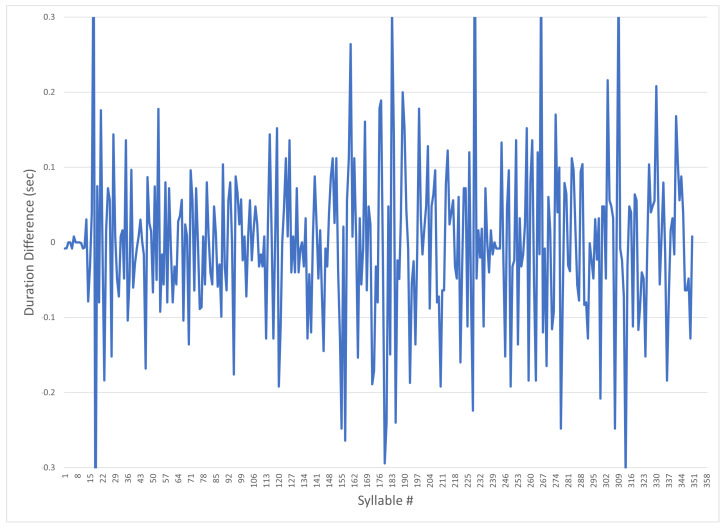
Praat post-manipulation delta of syllable duration.

The duration of the pauses before and after, and the durations of the voiced syllables differ by less than 10%, post-manipulation; however, this difference is audible, as the pace of the spoken words has varying timing—note that the values’ changes are very small for a large subjective effect on the spoken voice. In order to demonstrate the difference, the graph in Figure 12 shows the difference between the objectively analyzed monotonous audio file before and after the prosodic enhancement. The *y*-axis represents the seconds of difference between the source and the updated audio, and the *x*-axis shows each syllable number in its original sequence.

The largest differences between the original audio and that post-manipulation is in the intensity level, measured in decibels (dB). The prosodic version has almost double the dynamic range—the interquartile range of the original audio is 49.9–53.1 dB, whereas the range of the prosodic audio is from 61.8 to 67.8 dB. This change in dynamic range adds an element of realism to the monotonous speech, as real speech shifts in intensity as phrases are spoken. If both the input and output waveforms are overlaid with the axes shifted this becomes clearly visible: similarly to Figure 11, the shape of the original waveform’s intensity is visible, which demonstrates that the authentic voice is being retained.

This is significant because the decibel is a logarithmic measure of audio power in which the power output is doubled for every 3 dB. The overall volume being increased from a median 51 dB to a median 65 dB perhaps demonstrates the quiet original recording compared with the presentation format of the TED talks that formed the training of the LSTM—there is no requirement in a quiet studio to “speak to the back of the room” as in a presentation.

### 5.4. Ablation Study

**Figure 13:**
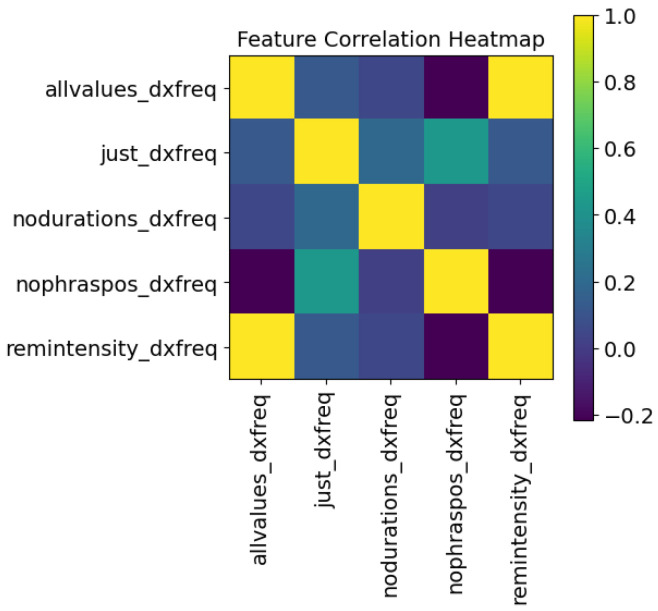
Ablated correlation “frequency“ model.

**Figure 14:**
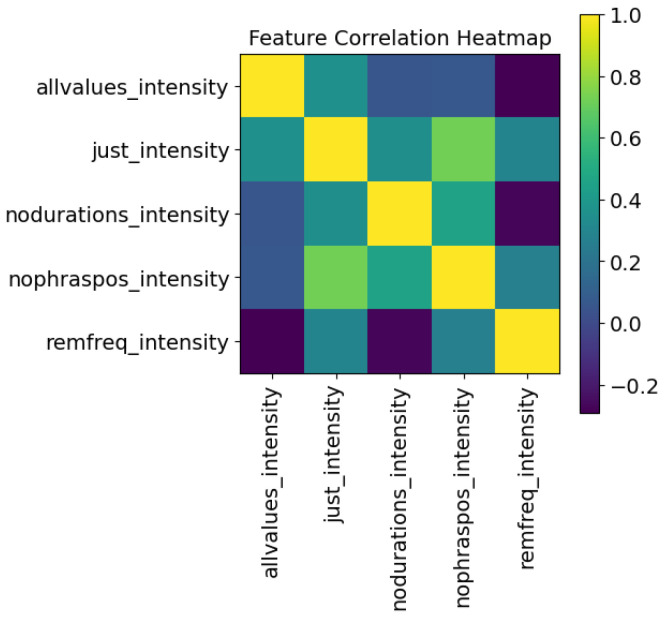
Ablated correlation “intensity“ model.

Recall that Figure 3 shows the correlation of the features and Section 4 discusses an approach to remove correlated features. An ablation study can be performed to better understand a model or to examine aspects of the contribution of different parts of the model. Usually, for an LSTM, specific types of gates are removed. In this study, as the LSTM was used as a predictive model, a different approach was used to achieve the same goal. In order to determine the causality of the features, an ablation of features on each LSTM was performed. The rows and columns in Figure 13 and Figure 14 are:

- “allvalues”—All of the features.
- “just_output”—Only the object of the LSTM, here frequency or intensity, and its delta values are utilized in training the model and prediction of the robotic sample.
- “nodurations”—All features related to duration (duration, pauses before and after) are removed.
- “nophraspos”—All features relates to the position of the syllable (phrase, phrase position, syllable#) are removed.
- “remobject”—In the two examples given, the intensity values are removed from the frequency LSTM, and the frequency values are removed from the intensity LSTM.

The values are removed prior to training and are also removed from the feature input of the robotic sample. The low correlation scores, compared to the full model of Figure 3, show that primarily the outputs are quite different when features are removed. The exceptions are that when removing the intensity values from the frequency LSTM model, the outputs have high correlation to utilizing all values; and in the intensity LSTM, removing phrase positioning is somewhat correlated to only using the intensity values and their deltas.

### 5.5. Qualitative Evaluation

As is common with studies of this type, an MOS test was conducted with 20 participants, each given 29 samples of audio ranging from one to six seconds, scored on a scale from 1 (robotic sounding) to 5 (authentic speech). The mean MOS score was 2.98, and the median score was 3. The results showed promise that with a small set of data an LSTM could be trained to make more natural-sounding, authentic speech. More varied sources for training data, and additional fine tuning of the hyperparameters, will improve upon these early findings.

## 6. Discussion

Despite advancements in the creation of computer-generated voices, there continues to be a clear audible distinction between the human recorded voice and computer synthetic voices. This research demonstrates that prosody can be added to recorded or synthetic voices for a more natural-sounding speech.

This paper has developed and explored a multivariate LSTM training model, experimenting with multiple inputs and single-prediction outputs, and multiple inputs with multiple-prediction outputs. Automation workflows to allow for large-scale calculations, with a primary focus on programming the multivariate elements and engaging the correct parameters, were implemented. Exporting the LSTM predictions into a Praat transformation demonstrated the validity of the prosodic improvement approach. Prosodic elements were added to make monotonic speech more authentic. This approach shows promise, with a median MOS of 3 out of 5, showing that the robotic sound samples were adjusted to become more human-like. The MOS is the standard mechanism to score objectively the subjective perception of audio, and a median score of 3—where 1 is robotic sounding and 5 is authentic human—suggests that the quality improved from its original robotic state. The score indicates that the transformation mitigated some of the robotic artifacts; the listeners’ scoring implies that they perceive the improvement but there remains scope for improvement. This work can be seen as a promising step towards enhancing the naturalness of the audio. This objectively demonstrates the addition of prosody. We intend to apply the approach to real-time continuous speech in future work.

Further exploration of the hyperparameters of the LSTM is also warranted, to fine-tune the model’s outputs, thus increasing realism. The use of notch filtering may also be effective in the input analysis stages as well as the output from the LSTM predictions to ensure the frequency remains within the mel-spectrum range of human auditory capacity. Additional non-prosodic sources will be obtained or generated, including those that are the result of excessive down sampling or excessive compression, to test the robustness of the algorithm. In addition to expanding the training set, further investigation into prosodic improvement of audio will be performed to identify the benefits in real situations such as low-bandwidth or corrupted transmissions.

## Figures and Tables

**Table 1:** Logic table of input variables and their targets for multivariate LSTM.

	δ Freq	δ Intensity	δ Duration	δ Pause Before	δ Pause After
phrase	Y	Y	Y	Y	Y
phrase_pos	Y	Y	Y	Y	Y
phrase_%	Y	Y	Y	Y	Y
δ freq	target	Y	Y	Y	Y
δ intensity	Y	target	Y	Y	Y
δ duration	Y	Y	target	Y	Y
δ pauseBefore	Y	Y	Y	target	Y
δ pauseAfter	Y	Y	Y	Y	target

## Data Availability

The data presented in this study are available on request from the corresponding author.

## Abbreviations

The following abbreviations are used in this manuscript:

ANN	Artificial neural network
DNN	Deep neural network
GAN	Generative adversarial neural network
HMM	Hidden Markov model
LSTM	Long short-term memory
MOS	Mean opinion score
PESQ	Perceptual evaluation of speech quality
RNN	Recurrent neural network
SAN	Storage area network
SVM	Support vector machine
TTS	Text-to-speech
